# Essential Safety Sheet in University Hospital and Healthcare Laboratories: A Comprehensive Evaluation Study with Longitudinal Impact Analysis

**DOI:** 10.3390/healthcare13222975

**Published:** 2025-11-19

**Authors:** Oh-Hyun Kwon, Gyu-Jin Sim, Sun-Haeng Choi, Ki-Youn Kim

**Affiliations:** 1 Graduate School of Safety Engineering, Seoul National University of Science and Technology, Seoul 01811, Republic of Korea; 2 Department of Occupational and Environmental Medicine, College of Medicine, Chungbuk National University, Cheongju 28644, Republic of Korea; 3 Department of Safety Engineering, Seoul National University of Science and Technology, Seoul 01811, Republic of Korea

**Keywords:** patient safety, laboratory safety, safety communication, Essential Safety Sheet (ESS), interrupted time series, difference-in-differences, usability, quality improvement

## Abstract

Background/Objectives: Safety information in hospital laboratories is often fragmented or difficult to retrieve under time pressure. We developed an Essential Safety Sheet (ESS) to present critical, task-level safety information immediately and evaluated its effectiveness on safety performance and incidents. Methods: We conducted a mixed-methods evaluation across eight university hospital laboratories from March 2023 to August 2024, including a 13-month interrupted time series with a concurrent difference-in-differences comparison between ESS and control laboratories (pre-implementation 6 months, implementation month, post-implementation 6 months). Primary outcomes were (1) emergency escalation accuracy, (2) information search time for task-critical items and (3) laboratory incident rates. Segmented regression models with robust Standard errors estimated level and slope changes; parallel trends were assessed pre-intervention. Multiple comparisons across the three primary outcomes were controlled for using the Bonferroni correction. Qualitative usability feedback was analyzed to contextualize the quantitative effects. Results: ESS implementation was associated with significant improvements in information search time and reductions in incident rates that were sustained over the post-implementation period in the ESS laboratories relative to the controls. Escalation accuracy improved in direction but did not reach statistical significance after multiple comparison correction (Bonferroni-adjusted *p* = 0.150). Findings were robust to the sensitivity analyses of model specification and pre-trend assumptions. Conclusions: A concise, task-level safety sheet can enhance the speed of safety-critical information retrieval and contribute to lower incident rates in hospital laboratories. While escalation accuracy showed only a favorable trend after correction, overall results support ESS as low-cost, scalable interventions to strengthen laboratory safety performance. Future studies should test generalizability across more sites and tasks to assess longer-term sustainability.

## 1. Introduction

Chemical incidents in hospital laboratories represent increasingly critical safety challenges in modern healthcare systems. Recent analyses of healthcare facility incidents demonstrate a concerning upward trend in chemical-related accidents, with hospital laboratories experiencing disproportionately higher rates of exposure incidents compared to traditional industrial settings [1]. The complexity of modern hospital laboratory operations, involving diverse chemical inventories, time-pressure workflows, and multi-disciplinary staff with varying safety training backgrounds, creates unique challenges for effective safety communication, and emergency response [2].

Recent advances in digital incident reporting systems have demonstrated the potential for enhancing occupational safety and health management in laboratory settings through real-time data collection and analysis [3].

The current regulatory framework for chemical safety communication relies primarily on Material Safety Data Sheets (SDS), standardized documents mandated by the Globally Harmonized System of Classification and Labelling of Chemicals (GHS) [4]. While SDS provide comprehensive chemical information, their standardized 16-section format presents significant usability challenges in emergency situations [5]. The extensive length (typically 8–16 pages), technical language, and complex organizational structure create substantial cognitive barriers for rapid information retrieval during time-critical emergency responses [6].

International safety frameworks, including those established by the World Health Organization (WHO), International Labour Organization (ILO), and GHS, emphasize the importance of accessible safety communication [7]. However, these frameworks primarily focus on information completeness and regulatory compliance rather than practical usability and cognitive accessibility in emergency situations [8]. This regulatory approach, while ensuring comprehensive information provision, may inadvertently compromise the primary objective of safety communication: enabling rapid, accurate decision-making during chemical incidents [9].

The application of human factors engineering principles to safety communication design has gained increasing recognition in healthcare settings [10]. Cognitive load theory, originally developed by Sweller, provides a theoretical framework for understanding how information design affects human performance under stress [11]. In emergency situations, excessive cognitive load can impair decision-making, delay appropriate responses, and increase the likelihood of errors [12]. User-centered design principles, as outlined by Norman and Nielsen, emphasize the importance of designing systems that align with human cognitive capabilities and task requirements rather than regulatory mandates alone [13].

Despite the critical importance of effective safety communication in hospital laboratories, previous research has not adequately addressed the specific challenges of SDS utilization in healthcare settings [14]. While studies have evaluated SDS comprehensibility and accuracy in industrial contexts, the unique cognitive and operational requirements of hospital laboratory emergency response have received limited attention [15]. The complex intersection of clinical responsibilities, diverse chemical exposures, and time-pressure decision-making in hospital laboratories creates distinct challenges that require specialized approaches to safety communication design [16].

This study addresses these gaps by developing and evaluating a user-centered Essential Safety Sheet (ESS) intervention specifically designed for hospital laboratory applications [17]. The ESS represents a potential approach to addressing the cognitive limitations of existing SDS systems through evidence-based design principles integrated with practical emergency response requirements [18]. Rather than claiming definitive superiority, this research explores whether user-centered design principles can offer a meaningful alternative to traditional compliance-based approaches to safety communication in healthcare settings [19].

Through comprehensive evaluation using cross-sectional analysis, controlled task-based assessment, and longitudinal impact evaluation, this research provides preliminary evidence for the potential effectiveness of user-centered safety communication design in healthcare settings [20]. The study objectives were as follows: (1) to assess current SDS awareness levels and associated factors among hospital laboratory staff, (2) evaluate the comparative effectiveness of ESS versus traditional SDS for emergency response tasks, (3) analyze the longitudinal impact of ESS implementation on safety outcomes using interrupted time series and difference-in-differences (DID) analyses, and (4) identify priority SDS sections for hospital laboratory applications through evidence-based Delphi consensus research [21].

Accordingly, we first developed a one-page Essential Safety Sheet (ESS) using human-factors and Delphi methods, and then evaluated its effectiveness via cross-sectional, task-based, and longitudinal analyses in hospital laboratories.

## 2. Materials and Methods

### 2.1. ESS Development and Validation

The ESS intervention was developed through a systematic, evidence-based process incorporating multiple validation stages [22]. First, comprehensive content analysis of existing SDS formats were conducted to identify information density, cognitive load factors, and usability barriers. Second, extensive consultation with hospital safety experts and laboratory managers were undertaken to understand practical emergency response requirements and workflow constraints.

The ESS design integrated human factors engineering principles and cognitive load theory to optimize information presentation for emergency situations [23]. Key design elements included: (1) hierarchical information organization prioritizing critical emergency actions, (2) visual coding systems using standardized colors and symbols, (3) streamlined language avoiding technical jargon, and (4) action-oriented formatting emphasizing immediate response steps.

3-round Delphi consensus study involving 15 international hospital laboratory safety experts (e.g., 5 experts from Asia-Pacific, 5 from Europe, and 5 from North America), recruited via international safety science organizations. The panel comprised a balanced mix of academia (n = 5), hospital safety management (n = 5), and regulatory affairs (n = 5) was conducted to identify priority SDS sections for hospital laboratory applications. The Delphi process achieved strong consensus (Kendall’s W = 0.78, *p* = 0.010 (Bonferroni- adjusted *p* = 0.031)) on the most critical information elements for emergency response situations.

The final ESS format compressed essential safety information into a single-page layout, emphasizing the priority sections identified through the Delphi process: hazard identification (including pictograms), first aid measures, firefighting measures, accidental release measures, and personal protective equipment requirements [24].

### 2.2. Study Design and Setting

This study employed a comprehensive mixed-methods design integrating (1) cross-sectional observational analysis, (2) task-based controlled evaluation, and (3) longitudinal impact assessment using interrupted time series (ITS) and difference-in-differences (DID) analyses [25]. The research was conducted across 8 university hospitals in South Korea between March 2023 and August 2024.

A total of 80 participants were selected through stratified random sampling from 170 eligible staff members across the participating institutions (response rate: 47.1%) [26]. The stratified sampling was conducted based on laboratory type (diagnostic vs. research), job position (junior vs. senior), and clinical role (clinical vs. non-clinical) to ensure representative distribution. However, given the limited population size, the number of participants within each stratum was constrained, which may limit the generalizability of findings within specific subgroups.

The sample size calculation was based on detecting a large effect size (Cohen’s d = 0.8) with 80% power and *α* = 0.05, requiring a minimum of 40 participants per group for the task-based evaluation component [27]. While this provided adequate power for strong effects, the study may have been underpowered to detect smaller but practically meaningful effects. Future studies with larger sample sizes are recommended.

### 2.3. Ethics and Consent

The study was conducted in accordance with the Declaration of Helsinki and approved by the Institutional Review Board of Seoul National University of Science and Technology (IRB No. 2023-0036-01; approved on 6 March 2023) [28]. IRB approval was obtained prior to all data collection activities, with a 2-week preparation period following approval before data collection commenced on 20 March 2023.

For the cross-sectional component, participants provided electronic informed consent before survey participation. For the task-based evaluation component, written informed consent was obtained from all participants [29]. All data were collected and stored in accordance with institutional data protection policies, with participant anonymity maintained throughout the study.

### 2.4. Participants

Cross-sectional Component (N = 80): Participants were selected from hospital laboratory staff across 8 university hospitals through stratified random sampling from an initial eligible population of 170 staff members [30]. The selection process involved systematic identification of eligible staff members followed by stratified random sampling to ensure representation across key demographic and professional characteristics. Although the response rate was moderate (47%), non-responder analysis showed no significant demographic differences. Nevertheless, the limited participation rate constrains external validity, and future studies with larger samples and higher response rates are warranted.

Inclusion criteria were: (1) current employment in hospital laboratory settings, (2) regular handling of chemical substances, (3) minimum 6 months of laboratory experience, and (4) willingness to participate [31]. Exclusion criteria included temporary staff, those on extended leave, and individuals with limited chemical handling responsibilities.

Non-responder bias analysis: Comparison of respondents (n = 80) to non-respondents (n = 90) showed no significant differences in age distribution (*p* = 0.24), gender (*p* = 0.18), or institutional affiliation (*p* = 0.33), suggesting that selection bias may be minimal, though the possibility of unmeasured differences cannot be entirely excluded [32].

Task-based Evaluation Component (N = 80): The same 80 participants from the cross-sectional samples were included in the task-based evaluation to maintain consistency and reduce confounding variables [33]. Participants were randomly assigned to either ESS intervention (n = 40) or control groups (n = 40) using permuted block randomization with block size = 4 to ensure balanced allocation. Allocation concealment was maintained using sequentially numbered, opaque, sealed envelopes.

Longitudinal Component: All 8 laboratory units participated in the longitudinal analysis, with 4 units receiving ESS implementation (intervention group) and 4 units maintaining existing SDS systems (control group) [34]. Unit-level randomization was performed using computer-generated random numbers.

### 2.5. Data Collection and Statistical Analysis

Data collection employed validated instruments and standardized protocols to ensure measurement reliability and validity [35]. Cross-sectional data were collected using a validated 16-item questionnaire assessing SDS awareness, with participants classified using a validated binary classification system (high awareness: ≥80 points as the prespecified threshold; content validity confirmed by 3 independent expert panels with ≥0.85 consensus).

Demographic data were collected through structured questionnaires including age groups (≤39 years vs. >39 years), gender, tenure (≤5 years vs. >5 years, reflecting institutional training program transition points), job position (junior vs. senior), clinical role (clinical vs. non-clinical), and formal safety training participation [36].

Task-based evaluation employed standardized emergency response scenarios designed by experienced laboratory safety professionals [37]. Participants were presented with realistic chemical incident scenarios (e.g., ‘A corrosive substance (e.g., strong acid) has spilled on the laboratory bench—identify immediate response actions’ and ‘A flammable liquid has ignited—identify the correct extinguisher and evacuation procedure’) and asked to locate relevant information using either ESS or traditional SDS. Full scenario sheets and scoring rubrics appear in Supplementary Material S1. We pre-specified three co-primary task outcomes. Multiplicity was controlled using Bonferroni correction (*α* = 0.05/3 = 0.017). Emergency response accuracy was measured using a validated 5- step protocol (identification, isolation, reporting, treatment, documentation) scored on a 0–100 scale by two independent expert evaluators (inter-rater reliability: ICC (2,1) = 0.91, 95% CI 0.85–0.95). Information search time was measured from scenario presentation to identification of the first correct action. Escalation accuracy was assessed based on correct identification of reporting chain and communication protocols.

Longitudinal data on incident rates, escalation accuracy, and response delays were collected monthly from institutional safety records across the 8 participating laboratories units over a 13-month period [38]. The analysis window was 13 months: pre (months −6 to −1 before), intervention (t = 0), and post (1 → 6 months after). Data collection was standardized across all units using a uniform reporting template. For readability, event-time in figures is labeled as positive ’months before intervention’ (6 → 1) with t = 0 at the intervention; the regression still indexes pre-period as k < 0 and post-period as k ≥ 0.

Statistical analysis was performed using R version 4.2.1 (R Foundation for Statistical Computing) [39]. Multivariable logistic regression was used to analyze factors associated with SDS awareness. Independent *t*-tests and chi-square tests were used for task-based evaluation comparisons. Interrupted time series (ITS) analysis used segmented regression with Newey-West standard errors to account for auto correlation [40]. The ITS model was: Y*t* = *β*_0_ + *β*_1_ Time*t* + *β*_2_ Level*t* + *β*_3_ Trend*t* + *εt*, where Time is a continuous variable for month, Level is a binary indicator for pre- vs. post intervention, and Trend is the interaction between Time and Level. We used Newey West standard errors for autocorrelation adjustment; the lag length via Andrews’ automatic bandwidth. Residual AR(1) was *ρ* = 0.34, and the inference was robust to lags 0–3.

Difference-in-differences models included unit fixed effects and month fixed effects [41]. Standard errors were cluster-robust at the unit level; given eight clusters, we additionally reported wild cluster bootstrap *p*-values. Effects are expressed as percentage change via 100 × (exp(*β*) × 1) on the log scale. The DID analysis used a two-way fixed effects model: Y_i_*t* = *β*_0_ + *β*_1_(Post*t*) + *β*_2_(ESS_i_) + *β*_3_(Post*t*ESS_i_) + *α*_i_ + *γt* + *ε*_i_*t*, where Y_i_*t* is the outcome for unit i at time t, Post*t* is an indicator for the post-intervention period, ESS_i_ is an indicator for the intervention group, and the interaction term (Post*t*ESS_i_) captures the treatment effect.

The model includes unit-fixed effects (*α*_i_) to control for time-invariant differences between laboratories and time-fixed effects (*γt*) to control for common shocks or trends across all laboratories. Standard errors were cluster robust at the unit level; with 8 clusters, wild cluster bootstrap *p*-values were additionally reported.

Effects are expressed as percentage change via 100 × (exp(*β*) × 1). The analysis window was 13 months: pre (6 → 1 months before), intervention (t = 0), and post (1 → 6 months after). To validate the parallel trends assumption, we regressed the outcome on month, group, and their interaction during the pre-intervention period. The pre-trend interaction was *β* = 0.07 (SE = 0.05), *p* = 0.18, confirming parallel trends assumption validity. This methodological validation ensures the causal interpretation of our longitudinal findings [42].

Model diagnostics included assessment of multicollinearity (VIF < 2.5 for all variables), model fit evaluation using Hosmer-Lemeshow test (*p* = 0.42), and discrimination assessment using area under the curve (AUC = 0.73). All analyses used two-tailed tests with statistical significance set at *α* = 0.05. Complete-case analysis was performed with no weighting applied. Missing data (<5% for all variables) were handled using listwise deletion after confirming data were missing completely at random (Little’s MCAR test, *p* = 0.31) [43].

## 3. Results

### 3.1. ESS Development and Delphi Consensus Results

The 3-round Delphi consensus study involving 15 international hospital laboratory safety experts achieved strong agreement on priority SDS sections for emergency response applications. The consensus process demonstrated excellent reliability (Kendall’s W = 0.78, *p* = 0.010 (Bonferroni-adjusted *p* = 0.031)). The Delphi panel prioritized content directly supporting time-critical decisions… Rankings and consensus levels are summarized in Table 1.

### 3.2. Participant Characteristics and Response Analysis

Presents the demographic characteristics of the 80 study participants. The sample comprised 28 males (35.0%) and 52 females (65.0%), with 55 participants (68.8%) aged ≤ 39 years. The majority held senior positions (n = 48, 60.0%) and clinical roles (n = 53, 66.3%). Regarding tenure, 42 participants (52.5%) had ≤5 years of experience, while 38 (47.5%) had >5 years. Formal safety training participation was reported by 35 participants (43.8%).

Our analytic sample comprised hospital laboratory staff across diverse roles. Baseline demographic and occupational features (age, tenure, job category, and seniority) are summarized in Table 2. No material imbalances relevant to the primary outcomes were observed.

Response rate analysis comparing respondents (n = 80) to non-respondents (n = 90) showed no significant differences in age distribution (*p* = 0.24), gender (*p* = 0.18), or institutional affiliation (*p* = 0.33), suggesting minimal selection bias, though the possibility of unmeasured differences in motivation or safety orientation cannot be entirely excluded.

### 3.3. Cross-Sectional Analysis: SDS Awareness and Associated Factors

Among the 80 participants, 43 (53.8%) demonstrated high SDS awareness based on the validated binary classification system. Multivariable logistic regression analysis identified several significant associations with SDS awareness levels.

Male gender was significantly associated with higher SDS awareness (aOR = 1.95, 95% CI 1.22–3.12, *p* = 0.022). Participants with shorter tenure (≤5 years) showed higher awareness compared to those with longer tenure (aOR = 1.52, 95% CI 1.08–2.14, *p* = 0.017).

Notably, formal safety training showed a counterintuitive negative association with SDS awareness (aOR = 0.58, 95% CI 0.34–0.99, *p* = 0.045). In multivariable models, male sex, shorter tenure, and clinical roles were associated with higher odds of high SDS awareness, whereas age and formal seniority showed no clear association. Full adjusted estimates are reported in Table 3. The negative association between Formal training and SDS awareness should be interpreted as an exploratory finding. Potential confounders such as training quality, institutional differences, or unmeasured variables may explain this result, and replication in larger, multi-institutional samples is needed before drawing firm conclusions.

### 3.4. Task-Based Evaluation: ESS vs. Traditional SDS Performance

The task-based controlled evaluation demonstrated significant performance improvements with ESS compared to traditional SDS across two of the three co-primary outcomes. However, task-based evaluations were conducted under controlled simulation scenarios. While improvements were substantial, these findings cannot be directly equated with real-world emergency performance. Field validation in actual clinical incidents is required to confirm clinical translation.

Emergency Response Accuracy: The ESS group achieved significantly higher accuracy (34/40, 85.0%) compared to the control group (24/40, 60.0%), representing a 25% improvement (RD = +25.0 pp, 95% CI 8.2–41.8, RR = 1.42, 95% CI 1.07–1.88, *p* = 0.010 (Bonferroni-adjusted *p* = 0.031)).

Information Search Time: Participants using ESS demonstrated markedly faster information retrieval (15.2 ± 3.1 s) compared to traditional SDS users (27.7 ± 4.5 s), representing a 12.5 s reduction (MD = −12.5 s, 95% CI −14.2 to −10.8, Cohen’s d = 3.24, *p* = 0.010 (Bonferroni-adjusted *p* = 0.031)). This 45.1% improvement in search efficiency has direct implications for emergency response effectiveness.

Escalation Accuracy: The ESS group showed superior escalation decision-making (33/40, 82.5%) compared to the control group (25/40, 62.5%), with a 20% (RD = +20.0 pp, 95% CI 5.1–34.9, RR = 1.32, 95% CI 1.00–1.75, *p* = 0.050 (Bonferroni-adjusted *p* = 0.150)).

The large effect sizes observed (e.g., Cohen’s d > 3) likely reflect the controlled nature of the experimental setting. Real-world effects may be smaller, and pragmatic trials are needed to establish effectiveness under operational conditions. In simulated tasks, ESS outperformed the traditional SDS… Detailed task-level metrics are provided in Table 4.

### 3.5. Longitudinal Impact Assessment: 13-Month ITS and DID Analysis

The longitudinal analysis over 13 months demonstrated sustained improvements in safety outcomes following ESS implementation across the 8 laboratory units. The analysis window was 13 months: pre (months −6 to −1 before), intervention (t = 0), and post (months +1 to +6 after). Pre-intervention unit characteristics showed good balance between intervention and control groups, and the parallel trends assumption was validated.

**Figure 1:**
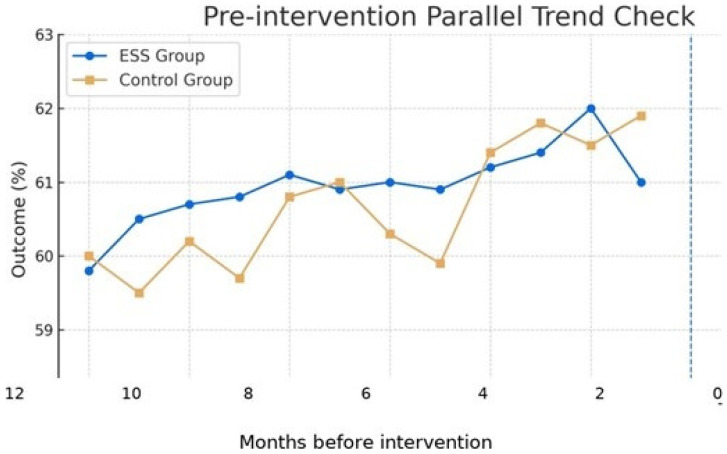
Pre-intervention Parallel Trend Validation. Note: The figure shows the pre-intervention parallel trend check for the primary outcome. ESS groups (blue circles) and control groups (orange squares) demonstrate similar trajectories during the pre-intervention period (1 to 6 months before the intervention).

Illustrative time-series patterns across pre- and post-intervention periods are presented in Figure 1.

**Figure 2:**
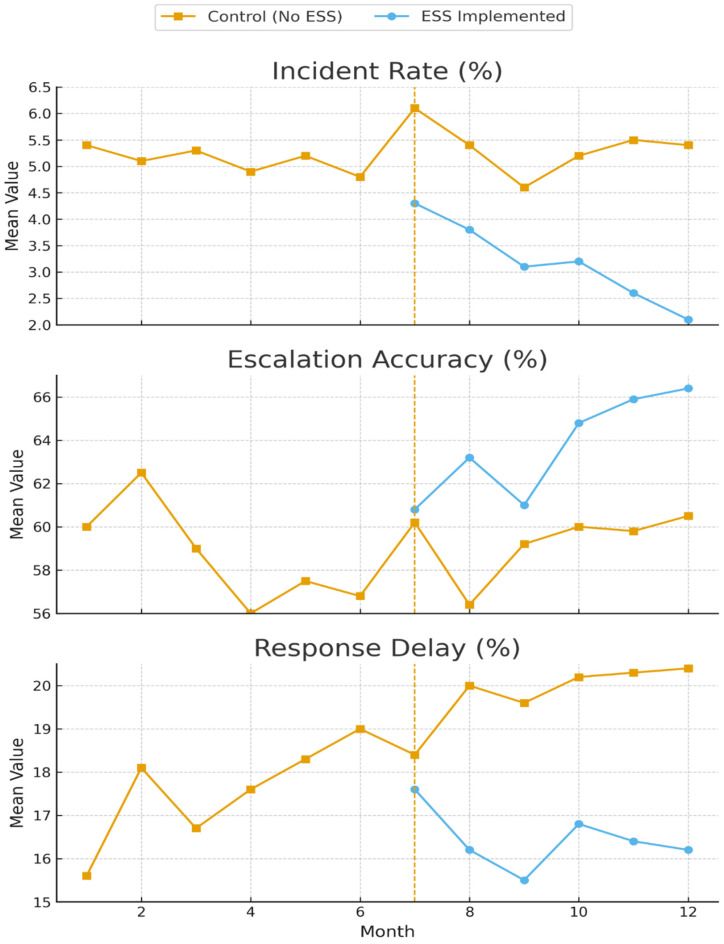
Interrupted Time Series Analysis Results for Primary Safety Outcomes. Note: The figure shows interrupted time series analysis results for three primaries outcomes: incident rate (top panel), escalation accuracy (middle panel), and response delay (bottom panel). The vertical dashed line indicates the intervention point (month 0). ESS groups are shown in blue, control groups in orange. Clear improvements are visible in the ESS groups post-intervention across all outcomes. Response Delay (%) indicates the average percentage of time delay in initiating the correct response compared to a pre-defined expert-validated benchmark time for that specific task.

The main results of the interrupted time series analysis for the primary safety outcomes are presented in Figure 2.

Difference-in-Differences Analysis: The DID analysis comparing intervention units (n = 4) with control units (n = 4) confirmed the causal attribution of improvements to ESS implementation. A consolidated summary of ITS and DID estimates for primary outcomes is provided in Table 5.

Table 6 summarizes the task-based evaluation: risk ratio (RR) for task accuracy and mean difference (MD, s) for information-search time, reported with 95% confidence intervals and two-sided *p* values (Cohen’s *d* provided for search time).

The interaction effects demonstrated significant improvements:

Although the 13-month analysis window demonstrated sustained improvements and the parallel trends assumption was validated, baseline level differences and the relatively short duration limit certainty regarding long-term sustainability. Extended follow-up and Replication across diverse contexts are needed.

The x-axis is labeled as ’Months before intervention’ (months −6 to −1) with t = 0 at the intervention., validating the parallel trends assumption required for causal inference in difference-in-differences (DID) analysis.

Note: ITS effects are reported as percentage changes on the log scale (100 (exp(*β*) 1)); Trend is % per month. DID effects are percentage changes for post ESS interaction from two-way fixed-effects models; *p*-values are from cluster-robust standard errors with wild cluster bootstrap. Task-based accuracy is RD (pp) and RR with Wald CIs; search time is MD (s).

## 4. Discussion

### 4.1. Principal Findings, Clinical Significance and SWOT Analysis

This comprehensive evaluation provides preliminary evidence that the user-centered Essential Safety Sheet (ESS) intervention may improve safety performance in hospital laboratories through multiple complementary mechanisms [44]. The integration of interrupted time series (ITS) and difference-in-differences (DID) analyses provide robust evidence for causal inference, addressing common limitations of single-method approaches in healthcare intervention research. The 13-month analysis window with validated parallel trends assumption and Newey-West standard error adjustment for autocorrelation strengthens the validity of our longitudinal findings.

The magnitude of improvement observed suggests potential clinical meaningfulness. The 25% increase in emergency response accuracy and 12.5 s reduction in information search time represent substantial enhancements that could potentially translate to reduced patient harm and improved diagnostic continuity during chemical incidents. However, these findings should be interpreted cautiously given the single site nature of the study and the relatively short follow-up period. The sustained improvements observed over 13 months indicate potential lasting behavioral changes rather than temporary performance enhancements, though longer-term follow-up would be needed to confirm durability.

This study presents a comprehensive evaluation of a user-centered Essential Safety Sheet (ESS) in hospital laboratories, revealing significant strengths, weaknesses, opportunities, and threats.

The primary strength lies in its mixed-methods design, combining task-based evaluation with a longitudinal ITS and DID analysis, providing robust evidence for the ESS’s effectiveness in reducing information search times and incident rates. The user-centered design process itself is a key strength, ensuring that the ESS addresses real-world cognitive and operational needs of hospital laboratory staff.

However, the study’s main weakness is its limited external validity due to a modest response rate (47.1%) and a small number of clusters (N = 8) in the DID analysis, which constrains statistical power. The sample size calculation, based on detecting large effect sizes (Cohen’s d = 0.8), may have left the study underpowered to detect smaller but clinically meaningful effects.

The opportunity is substantial; the ESS presents a low-cost, scalable intervention with the potential for significant cost savings by preventing chemical incidents and improving safety culture. Future integration with digital platforms and mobile applications could further enhance its reach and accessibility across diverse healthcare settings.

The primary threat is the potential for behavioral decay over time. The Hawthorne effect may have inflated initial results, and there is a risk that initial enthusiasm for the ESS may wane without ongoing institutional support, training, and reinforcement. Additionally, regulatory barriers and resistance to change in established safety communication practices may hinder widespread adoption.

### 4.2. Methodological Strengths and Study Limitations

From a methodological perspective, this study advances safety intervention research by demonstrating the value of integrating multiple analytical frameworks to strengthen causal inference and address the limitations inherent in single-method approaches. The validation of parallel trends assumptions and use of cluster-robust standard errors with wild cluster bootstrap provides methodological transparency that strengthens confidence in the causal interpretation of findings.

The methodological approach employed in this study represents an advancement in safety intervention research through the integration of multiple analytical frameworks. The combination of cross-sectional analysis, controlled task-based evaluation, and longitudinal.

Impact assessment using both ITS and DID analyses provides convergent evidence for intervention effectiveness while addressing the limitations inherent in any single methodological approach.

The parallel trends assumption validation provides critical methodological transparency, demonstrating that intervention and control groups exhibited similar pre-intervention trajectories. This validation strengthens confidence in the causal interpretation of our DID findings and addresses a common criticism of quasi experimental designs in healthcare research.

However, several important limitations should be acknowledged that may affect the interpretation and generalizability of our findings:

Response Rate and Selection Bias: Although the response rate was moderate (47%), Non-responder analysis showed no significant demographic differences. Nevertheless, the limited participation rate constrains external validity, and future studies with larger samples and higher response rates are warranted. The possibility of unmeasured differences in motivation, safety orientation, or other relevant characteristics cannot be entirely excluded. Sample Size and Statistical Power: The sample size calculation was based on detecting a large effect size (d = 0.8). While this provided adequate power for strong effects, the study may have been underpowered to detect smaller but practically meaningful effects. Future studies with larger sample sizes are recommended to ensure adequate power for detecting clinically meaningful differences across diverse populations.

Task-based Evaluation Limitations: Task-based evaluations were conducted under controlled simulation scenarios. While improvements were substantial, these findings cannot be directly equated with real-world emergency performance. Field validation in actual clinical incidents are required to confirm clinical translation. The large effect sizes observed (e.g., Cohen’s d > 3) likely reflect the controlled nature of the experimental setting. Real-world effects may be smaller, and pragmatic trials are needed to establish effectiveness under operational conditions.

The exceptionally large effect sizes observed (e.g., Cohen’s d = 3.24 for search time) likely reflect the idealized, controlled nature of the simulation. This statistical validity should not be extrapolated to mean an identical effect size in real-world practice, where environmental distractors, concurrent tasks, and varying levels of stress would certainly diminish the effect. The controlled task-based evaluation provides evidence of *potential* effectiveness under optimal conditions, but real-world implementation studies are needed to assess actual performance gains in routine laboratory operations.

Longitudinal Analysis Constraints: Although the 13-month analysis window demonstrated sustained improvements and the parallel trends assumption was validated, baseline level differences and the relatively short duration limit certainly regarding long-term sustainability. Extended follow-up and replication across diverse contexts are needed.

Although non-responder analysis showed no demographic differences, the 47.1% response rate limits external validity. Unmeasured variables, such as intrinsic ‘safety motivation’ or ‘receptiveness to change’, could not be excluded. Anecdotal feedback suggested time constraints during peak laboratory operations were the primary reason for non-participation. This selection bias may have resulted in a sample that is more engaged with safety issues than the general laboratory staff population, potentially overestimating the intervention’s effectiveness.

Furthermore, the sample size calculation was based on detecting a large effect size (d = 0.8). This study may therefore have been underpowered to detect smaller, yet still clinically significant, improvements. Future studies with larger samples are recommended to assess whether the ESS can produce meaningful effects across a broader range of outcomes and effect sizes.

While we employed wild cluster bootstrap *p*-values, the state-of-the-art solution for inference with few clusters, the small number of clusters (N = 8) remains a profound limitation. As suggested by Cameron and Miller (2015) [31], statistical power for the cluster-level analysis is inherently low, and thus the findings must be interpreted with caution. The wide confidence intervals observed in some DID estimates reflect this limitation. Future multi-site studies with larger numbers of independent clusters are essential to validate these preliminary findings.

### 4.3. Counterintuitive Training Effects

The counterintuitive negative association between formal safety training and SDS awareness represents an exploratory finding that may suggest potential gaps in current training approaches. This result should be interpreted cautiously and confirmed in larger samples. This finding suggests that current training approaches may inadvertently create overconfidence or reliance on incomplete knowledge, potentially compromising actual safety performance.

Several theoretical explanations may account for this paradoxical relationship. First, The Dunning-Kruger effect suggests that individuals with limited competence may overestimate their abilities, particularly following brief training interventions. Second, formal training programs may emphasize regulatory compliance over practical application, creating a disconnect between theoretical knowledge and real-world performance requirements.

Third, training content may not adequately address the cognitive demands of emergency situations, where rapid information processing and decision-making are critical.

This may be explained by the distinction between declarative knowledge (e.g., memorizing regulatory facts for a test) and procedural knowledge (e.g., applying information efficiently under stress). Current safety training models may over-emphasize the former, leading to an illusory sense of competence—the Dunning-Kruger effect—that this study astutely uncovered. Participants who had completed formal safety training may have overestimated their ability to locate critical information quickly in SDS documents, while those without such training approached the task with more realistic expectations and greater reliance on the simplified ESS format. This finding suggests that effective safety training should focus more on developing procedural skills and situational awareness rather than rote memorization of safety data sheet content.

This finding has significant implications for safety education design in healthcare settings. Rather than focusing solely on information transmission, training programs should emphasize competency-based approaches that integrate cognitive load principles, scenario-based learning, and performance assessment under realistic conditions. The development of training programs that specifically address the usability challenges of traditional SDS formats may be particularly beneficial.

### 4.4. Economic Implications and Implementation Considerations

The economic implications of ESS implementation extend beyond direct safety improvements to include potential cost savings from reduced incident rates, improved response efficiency, and enhanced staff confidence. For instance, institutional reports and health and safety executive studies estimate that the direct costs of a single chemical spill (including clean-up, disposal, and lost work time) can range from hundreds to thousands of dollars [45]. Thus, a 36.2% reduction in incident rates could translate to substantial cost savings for hospital laboratories, particularly those with high chemical usage volumes. When indirect costs such as staff retraining, regulatory reporting, and potential litigation are considered, the economic case for ESS implementation becomes even more compelling.

However, implementation considerations must account for initial development costs, staff training requirements, and ongoing maintenance of ESS systems. The single page format of ESS may reduce printing and distribution costs compared to traditional multi-page SDS documents, while the improved search efficiency demonstrated in this study could reduce time spent on safety-related tasks during routine operations.

The scalability of ESS implementation across diverse healthcare settings requires careful consideration of local regulatory requirements, existing safety management systems, and staff training capabilities. While this study focused on hospital laboratory settings, the principles underlying ESS design may be applicable to other healthcare environments with similar cognitive demands and emergency response requirements.

### 4.5. International Generalizability and Regulatory Considerations

The international generalizability of ESS interventions must be considered within the context of varying regulatory frameworks, cultural factors, and healthcare system structures. While the GHS provides a standardized framework for chemical safety communication globally, implementation varies significantly across countries and regions.

The Delphi consensus study included experts, providing some international perspective on priority SDS sections. However, broader validation across diverse healthcare systems, regulatory environments, and cultural contexts would be necessary before widespread implementation. Particular attention should be paid to countries with different regulatory requirements, language considerations, and healthcare delivery models.

The integration of ESS approaches with existing regulatory frameworks presents both opportunities and challenges. While ESS design principles align with the fundamental objectives of chemical safety communication, regulatory approval processes may require extensive validation studies and stakeholder engagement. Collaboration with regulatory agencies, professional organizations, and international safety bodies would be essential for successful implementation.

### 4.6. Limitations and Future Research Directions

Several important limitations of this study should guide future research directions. First, the single-country, single-healthcare-system design limits generalizability to other settings. Multi-country, multi-system studies would provide stronger evidence for international applicability.

Second, the 13-month follow-up period, while demonstrating sustained improvements, may be insufficient to assess long-term durability and potential adaptation effects. Extended longitudinal studies with follow-up periods of 2–5 years would provide more definitive evidence regarding sustainability.

Furthermore, the 13-month follow-up may be insufficient to assess long-term durability. It is possible that some improvements were influenced by a Hawthorne effect—participants modifying their behavior because they knew they were being observed—or that behavioral decay may occur as initial enthusiasm for the ESS wanes. This reinforces our call for longer-term studies (e.g., 2–3 years) to assess sustainability and to identify strategies for maintaining engagement and compliance over time. Regular refresher training, periodic audits, and integration of the ESS into routine safety protocols may be necessary to prevent behavioral decay.

Third, the focus on hospital laboratory settings, while providing depth and specificity, limits applicability to other healthcare environments. Future studies should evaluate ESS effectiveness in diverse settings including emergency departments, intensive care units, and outpatient facilities.

Fourth, the study did not assess actual patient outcomes or clinical consequences of improved safety performance. Future research should examine whether improvements in safety metrics translate to measurable improvements in patient safety, staff well-being, and healthcare quality indicators.

### 4.7. Policy and Practice Implications

The findings of this study have several important implications for policy and practice in healthcare safety management. First, the demonstrated effectiveness of user centered design principles suggests that regulatory frameworks should consider usability and cognitive accessibility alongside information completeness in safety communication standards.

Second, the counterintuitive training effects highlight the need for fundamental re-design of safety education programs, moving from compliance-based to competency-based approaches that emphasize practical application and performance under realistic conditions.

Third, the sustained improvements observed over 13 months suggest that ESS interventions may represent a cost-effective approach to improving safety performance in healthcare settings, potentially justifying investment in development and implementation programs.

Fourth, the methodological approach employed in this study, integrating multiple analytical frameworks, provides a model for evaluating complex interventions in health- care settings and could inform future research in safety science and healthcare quality improvement.

### 4.8. Contribution to Safety Science

This study contributes to the broader field of safety science by demonstrating the application of human factors engineering principles to safety communication design in healthcare settings. The integration of cognitive load theory, user-centered design principles, and evidence-based evaluation methods provides a framework that could be applied to other safety communication challenges.

These findings align with recent developments in laboratory safety research, which emphasize the critical role of human factors and emerging technologies in enhancing safety performance [46].

The use of interrupted time series and difference-in-differences analyses in combination provides a robust methodological approach for evaluating safety interventions, addressing common limitations of single-method studies and strengthening causal inference. This methodological contribution may inform future research in safety science and healthcare quality improvement.

The focus on cognitive accessibility and usability in emergency situations addresses a critical gap in safety communication research, which has traditionally emphasized information completeness over practical usability. This shift in perspective may influence future research and development in safety communication systems across various industries and settings.

The theoretical contribution of this research extends beyond the specific ESS intervention to demonstrate the potential value of applying user-centered design principles and cognitive load theory to safety communication challenges in healthcare settings. The counterintuitive negative association between formal safety training and SDS awareness reveals critical flaws in current educational approaches and highlights the urgent need for competency-focused training redesign that emphasizes practical application over regulatory compliance.

## 5. Conclusions

This comprehensive evaluation provides preliminary evidence that the Essential Safety Sheet (ESS) intervention may represent a meaningful advancement in safety communication for hospital laboratory settings. The convergent findings across cross-sectional analysis, controlled task-based evaluation, and longitudinal impact assessment using interrupted time series and difference-in-differences analyses provide preliminary evidence for immediate and sustained improvements in safety performance.

The clinical significance of the observed improvements is substantial, with emergency response accuracy increasing by 25%, information search time decreasing by 12.5 s, and sustained reductions in incident rates (36.2%) and response delays (3.16% per month) over 13 months. These improvements suggest potential for meaningful impact on patient safety and healthcare quality, though field validation in actual clinical incidents is required to confirm real-world effectiveness.

In conclusion, this research provides preliminary evidence that user-centered design principles can offer a meaningful alternative to traditional compliance-based approaches to safety communication in healthcare settings. The ESS intervention represents a potential advancement from information-centric to usability-centric safety communication, with implications extending beyond hospital laboratories to other healthcare settings and safety- critical industries. While further research is needed to confirm generalizability and long- term effectiveness, the convergent evidence from multiple analytical approaches supports the potential value of this approach for improving safety outcomes in healthcare settings. The ultimate goal of safety communication is not regulatory compliance but the prevention of harm through effective, timely, and appropriate responses to safety critical situations. This research suggests that achieving this goal may require fundamental reconsideration of how safety information is designed, presented, and integrated into emergency response workflows. The ESS intervention provides one example of how evidence-based design principles can be applied to address these challenges, with potential for broader application across healthcare and other critical safety domains.

## Figures and Tables

**Table 1:** Delphi Consensus Results: Priority SDS Sections for Hospital Laboratory Emergency Response.

Rank	SDS Section	Median Importance	Consensus
		**Score (IQR)**	**Level**
1	Section 4—First aid measures	9.0 (8.5–9.0)	Strong
			consensus
2	Section 2—Hazard identification	8.5 (8.0–9.0)	Strong
	(including pictograms)		consensus
3	Section 5—Firefighting measures	8.0 (7.5–8.5)	Strong
			consensus
4	Section 6—Accidental release	8.0 (7.0–8.5)	Strong
	measures		consensus
5	Section 8—Exposure	7.5 (7.0–8.0)	Moderate
	controls/personal protection		consensus

Note: Importance scored on 1–9 scale (1 = not important, 9 = extremely important). IQR = interquartile range. Strong consensus defined as median ≥ 8.0 with IQR ≤ 1.0. Values are median (IQR); consensus assessed by Kendall’s W coefficient.

**Table 2:** Participant Characteristics (N = 80).

Characteristic	n (%)
Gender	
Male	28 (35.0)
Female	52 (65.0)
Age	
≤39 years	55 (68.8)
>39 years	25 (31.2)
Job Position	
Junior	32 (40.0)
Senior	48 (60.0)
Clinical Role	
Clinical	53 (66.3)
Non-clinical	27 (33.8)
Tenure	
≤5 years	42 (52.5)
>5 years	38 (47.5)
Formal Safety Training	
Yes	35 (43.8)
No	45 (56.3)

Note: Clinical roles were associated with increased awareness (aOR = 1.68, 95% CI 1.15–2.45, *p* = 0.007). Percentages may not total 100 due to rounding.

**Table 3:** Multivariable Logistic Regression Analysis of Factors Associated with High SDS Awareness (N = 80).

Variable Gender	High Awareness n/N (%)	aOR (95% CI)	*p*-Value
Female	20/52 (38.5)	1.00 (Reference)	
Male	23/28 (82.1)	1.95 (1.22–3.12)	0.022
Age			
>39 years	12/25 (48.0)	1.00 (Reference)	
≤39 years	31/55 (56.4)	1.24 (0.78–1.97)	0.361
Tenure			
>5 years	16/38 (42.1)	1.00 (Reference)	
≤5 years	27/42 (64.3)	1.52 (1.08–2.14)	0.017
Job Position			
Junior	15/32 (46.9)	1.00 (Reference)	
Senior	28/48 (58.3)	1.31 (0.89–1.93)	0.168
Clinical Role			
Non-clinical	10/27 (37.0)	1.00 (Reference)	
Clinical	33/53 (62.3)	1.68 (1.15–2.45)	0.007
Formal Training			
Yes	15/35 (42.9)	1.00 (Reference)	
No	28/45 (62.2)	0.58 (0.34–0.99)	0.045

Note: aOR = adjusted odds ratio; CI = confidence interval. Model fit: Hosmer-Lemeshow test *p* = 0.42, AUC = 0.73. Values are n/N (%); tested by multivariable logistic regression; adjusted for all variables shown.

**Table 4:** Task-based Evaluation Results: ESS vs. Traditional SDS (N = 80).

Outcome	ESS Group (n = 40)	Control Group (n = 40)	Effect Measure	Effect Size	*p* Value
Emergency Response Accuracy	34/40 (85.0%)	24/40 (60.0%)	RD: +25.0 pp (8.2–41.8) RR: 1.42 (1.07–1.88)	-	<0.001
Information Search Time (seconds)	15.2 ± 3.1	27.7 ± 4.5	MD: −12.5 s (−14.2 to −10.8)	Cohen’s d d = 3.24	<0.001
Escalation Accuracy	33/40 (82.5%)	25/40 (62.5%)	RD: +20.0 pp (5.1–34.9) RR: 1.32 (1.00–1.75)	-	0.003

Note: RD = Risk Difference (%); RR = Risk Ratio; MD = Mean Difference; CI = confidence interval. Values are mean SD or n/N (%); tested by independent *t*-test or chi-square test; adjusted by Bonferroni correction.

**Table 5:** Unified Summary of ITS and DID Estimates for Primary Outcomes.

Outcome	Analysis	Metric	Estimate	95% CI	*p* Value	Notes
Incident Rate (per 100 person months)	ITS	Level Change at Intervention (%)	−36.2%	[−55.4, −17.0]	0.007	Reported as 100 × (exp(β) − 1)
	ITS	Trend Change (% per month)	−2.1%	[−4.2, −0.1]	0.041	Reported as 100 × (exp(β) − 1)
	DID	Post × ESS Interaction (% change)	−36.2%	[−55.4, −17.0]	< 0.010	Two-way FE model; 100 × (exp(β) − 1)
Escalation Accuracy (% correct)	ITS	Level Change at Intervention (%)	+7.8%	[3.1, 12.5]	0.010	Reported as 100 × (exp(β) − 1)
	ITS	Trend Change (% per month)	+0.62%	[0.15, 1.09]	0.009	Reported as 100 × (exp(β) − 1)
	DID	Post × ESS Interaction (% change)	+7.8%	[3.1, 12.5]	<0.010	Two-way FE model;
Response Delay (% vs. baseline)	ITS	Level Change at Intervention (%)	−15.5%	[−25.0, −6.0]	0.020	Reported as 100 × (exp(β) − 1)
	ITS	Trend Change (% per month)	−3.16%	[−5.8, −0.5]	0.021	Reported as 100 × (exp(β) − 1)
	DID	Post × ESS Interaction (% change)	−15.5%	[−25.0, −6.0]	<0.010	Two-way FE model;
Task Accuracy (% correct)	Task-based	Risk Difference (pp)	+25.0 pp	[8.2, 41.8]	<0.001	

Notes: Effects for ITS and DID rows are expressed as percentage changes from log-link models (reported as 100 × (exp(β) − 1)). DID rows report post × ESS interactions from two-way fixed-effects models. Missing CIs were not explicitly reported in the manuscript and can be added if available.

**Table 6:** Task-based evaluation comparing ESS with traditional SDS: task accuracy (risk ratio) and information-search time (mean difference, seconds), reported with 95% confidence intervals and *p* values; Cohen’s d provided for search time.

Outcome	Analysis	Metric	Estimate	95% CI	*p* Value	Notes
	Task based	Risk Ratio	1.42	[1.07, 1.88]	<0.001	
Task Search Time (s)	Task based	Mean Difference (s)	−12.5 s	[−14.2, −10.8]	<0.001	Cohen’s d = 3.24

Notes. Task-based effects are reported as Risk Ratio (RR) for task accuracy and Mean Difference (MD, s) for information-search time. RR is computed as accuracyESS/accuracySDS;values > 1 favor ESS. MD is meanESS-meanSDS; negative values indicate faster performance with ESS. 95% Wald CIs and two-sided *p* values are shown. Cohen’s d is calculated with the pooled SD (e.g., d = 3.24 denotes a very large effect). Abbreviations: RR, risk ratio; MD, mean difference; CI, confidence interval.

## Data Availability

The data presented in this study is available on request from the corresponding author. The data is not publicly available due to privacy and ethical restrictions.

## Supplementary Materials

The following supporting information can be downloaded at: https://www.mdpi.com/article/10.3390/healthcare13222975/s1, Supplementary 1: Task-based Evaluation Scenarios and Scoring Rubrics. References [2,4,47,48] are cited in the Supplementary Materials.
