# Supplementary material for: Essential Safety Sheet in University Hospital and Healthcare Laboratories: A Comprehensive Evaluation Study with Longitudinal Impact Analysis
Source: Healthcare (Basel). 2025 Nov 19;13(22):2975. doi: 10.3390/healthcare13222975 (PMC12652163; doi:10.3390/healthcare13222975)
Supplement: Supplementary file 1 [file healthcare-13-02975-s001.zip › healthcare-3905452-supplementary.pdf]

Supplementary 1. Task-based Evaluation Scenarios and Scoring Rubrics

This appendix provides detailed descriptions of the four standardized emergency response scenarios used in the task-based evaluation component of this study. Each scenario was designed by experienced laboratory safety professionals to represent realistic chemical incidents commonly encountered in hospital laboratory settings. All scenarios were pilot-tested with 5 laboratory staff members not included in the main study to ensure clarity, realism, and appropriate difficulty level.

Scenario 1: Corrosive Splash (Strong Acid)

Situation Description

A 500 mL bottle of concentrated sulfuric acid (98%, H_2SO_4) has been accidentally knocked over during routine laboratory work. Approximately 200 mL of the acid has spilled onto the laboratory bench surface, with minor splashing onto the floor. No personnel exposure has occurred, but the spill is actively spreading and producing visible fumes. The laboratory is occupied by 3 staff members at the time of the incident.

Chemical Information Provided

- **Substance**: Sulfuric acid, concentrated (98%)

- **GHS Classification**: Corrosive to metals (Category 1), Skin corrosion (Category 1A), Serious eye damage (Category 1)

- **Physical State**: Clear, colorless to slightly yellow liquid

- **Odor**: Odorless or slight odor

Task Requirements (Time-limited)

Task 1: Identify First Aid Measures (Time limit: 30 seconds)
Instructions: "Using the provided safety information sheet, identify the correct first aid measures for skin contact with this substance."

Correct Response Elements (must identify at least 3 of 4):

1. Immediately remove contaminated clothing

2. Rinse affected area with copious amounts of water for at least 15 minutes

3. Do NOT attempt to neutralize with chemicals

4. Seek immediate medical attention

Task 2: Locate Personal Protective Equipment Requirements (Time limit: 20 seconds)
Instructions: "Identify the minimum PPE required for personnel responding to this spill."

Correct Response Elements (must identify all 4):

1. Chemical-resistant gloves (nitrile or neoprene)

2. Safety goggles or face shield

3. Laboratory coat or chemical-resistant apron

4. Closed-toe shoes (chemical-resistant preferred)

Task 3: Determine Spill Response Procedure (Time limit: 40 seconds)
Instructions: "Outline the immediate response actions required for this spill situation."

Correct Response Elements (must identify at least 4 of 6):

1. Evacuate non-essential personnel from immediate area

2. Ensure adequate ventilation (activate fume hood if applicable)

3. Wear appropriate PPE before approaching spill

4. Contain spill using absorbent materials (acid-neutralizing absorbent preferred)

5. Notify laboratory supervisor/safety officer

6. Do NOT pour water directly onto concentrated acid

Scoring Rubric
|------------------|-------|----------|

Information Search Time Measurement
- **Start point**: Moment scenario description and Instructions are presented to participant

- **End point**: Moment participant identifies first correct action element (verified by evaluator)

- **Recording method**: Digital timer with millisecond precision

- **Acceptable range**: 10-60 seconds (based on pilot testing)

Scenario 2: Flammable Spill (Solvent Ignition)

Situation Description
During a routine extraction procedure, a 250 mL bottle of acetone has been accidentally knocked over near a hot plate that was inadvertently left on. Approximately 150 mL of

acetone has spilled onto the laboratory bench and has ignited, creating a small but spreading fire (approximately 30 cm diameter). The fire has not yet spread to other materials, but several paper towels and plastic containers are within 50 cm of the flames. One laboratory staff member is within 2 meters of the fire.

Chemical Information Provided

- **Substance**: Acetone (2-Propanone, $(CH_3)_2CO$)

- **GHS Classification**: Flammable liquid (Category 2), Eye irritation (Category 2A)

- **Flash Point**: -20°C (-4°F)

- **Auto-ignition Temperature**: 465°C (869°F)

- **Flammable Limits**: LEL 2.5%, UEL 12.8%

Task Requirements (Time-limited)

Task 1: Identify Immediate Fire Response Actions (Time limit: 25 seconds)

Instructions: "Using the provided safety information sheet, identify the immediate actions required for this fire situation."

Correct Response Elements (must identify at least 4 of 5):

1. Activate fire alarm if fire cannot be immediately controlled

2. Evacuate personnel from immediate danger zone

3. Close containers of flammable materials if safe to do so

4. Turn off ignition source (hot plate) if safe to approach

5. Do NOT use water to extinguish solvent fire

Task 2: Identify Correct Fire Extinguisher Type (Time limit: 20 seconds)

Instructions: "Identify the appropriate fire extinguisher type for this incident."

Correct Response Elements (must identify at least 2 of 3):

1. Class B fire extinguisher (for flammable liquids)

2. CO_2 or dry chemical extinguisher

3. Do NOT use water-based extinguisher

Task 3: Determine Evacuation and Reporting Procedures (Time limit: 30 seconds)

Instructions: "Outline the evacuation and reporting procedures for this incident."

Correct Response Elements (must identify at least 3 of 5):

1. Evacuate all personnel if fire spreads beyond initial containment

2. Close laboratory door when evacuating (do not lock)

3. Activate building fire alarm system

4. Call emergency services (fire department)

5. Report to designated assembly point and account for all personnel

Scoring Rubric
|------------------|-------|----------|

Information Search Time Measurement
- **Start point**: Moment scenario description is presented

- **End point**: Moment participant correctly identifies fire extinguisher type

- **Recording method**: Digital timer with millisecond precision

- **Acceptable range**: 8-45 seconds (based on pilot testing)

Scenario 3: Carcinogen Exposure (Controlled Vial Breach)

Situation Description
A sealed vial containing 10 mL of formaldehyde solution (37% concentration, formalin) has developed a crack during storage, resulting in a slow leak. The leak was discovered during routine inventory check. Approximately 2-3 mL of solution has leaked onto the storage shelf surface. The storage area is a closed cabinet with limited ventilation. No personnel exposure has been confirmed, but the characteristic pungent odor is detectable when the cabinet is opened.

Chemical Information Provided
- **Substance**: Formaldehyde solution (37% in water, with methanol as stabilizer)

- **GHS Classification**: Acute toxicity (Category 3), Skin corrosion (Category 1B), Carcinogenicity (Category 1B), Skin sensitization (Category 1)

- **Exposure Limits**: OSHA PEL: 0.75 ppm (TWA), 2 ppm (STEL)

- **Physical State**: Clear, colorless liquid with pungent odor

Task Requirements (Time-limited)

Task 1: Identify Exposure Control Measures (Time limit: 35 seconds)
Instructions: "Using the provided safety information sheet, identify the exposure control measures required for handling this situation."

Correct Response Elements (must identify at least 4 of 6):

1. Ensure adequate ventilation before approaching (open fume hood or activate exhaust)

2. Wear chemical-resistant gloves (nitrile minimum)

3. Wear safety goggles and face shield

4. Wear respiratory protection if ventilation is inadequate (organic vapor cartridge minimum)

5. Wear laboratory coat or disposable protective suit

6. Minimize time in exposure area

Task 2: Identify Health Hazard Information (Time limit: 30 seconds)
Instructions: "Identify the primary health hazards associated with exposure to this substance."

Correct Response Elements (must identify at least 3 of 5):

1. Suspected human carcinogen (IARC Group 1)

2. Causes severe skin burns and eye damage

3. May cause allergic skin reaction (sensitization)

4. Toxic if inhaled, swallowed, or absorbed through skin

5. Irritation to respiratory tract

Task 3: Determine Waste Disposal Procedures (Time limit: 25 seconds)
Instructions: "Identify the correct disposal procedure for contaminated materials."

Correct Response Elements (must identify at least 3 of 4):

1. Collect contaminated materials in designated hazardous waste container

2. Label container with chemical name and hazard classification

3. Do NOT dispose in regular trash or sink

4. Follow institutional hazardous waste disposal protocols

Scoring Rubric
|------------------|-------|----------|

Information Search Time Measurement
- **Start point**: Moment scenario description is presented

- **End point**: Moment participant identifies carcinogenic classification

- **Recording method**: Digital timer with millisecond precision

- **Acceptable range**: 12-50 seconds (based on pilot testing)

Scenario 4: Oxidizer Release (Small Leak)

Situation Description
A 500 mL bottle of hydrogen peroxide solution (30% concentration, H_2O_2) stored in a chemical refrigerator has developed pressure buildup due to decomposition, causing the cap to loosen and release approximately 50 mL of solution onto the refrigerator shelf. The solution has come into contact with paper labels and cardboard packaging materials, which are showing signs of discoloration. The refrigerator door was opened by a staff member who noticed the leak and unusual odor.

Chemical Information Provided
- **Substance**: Hydrogen peroxide solution (30% in water)

- **GHS Classification**: Oxidizing liquid (Category 2), Acute toxicity (Category 4), Skin corrosion (Category 1A), Serious eye damage (Category 1)

- **Decomposition**: Decomposes to oxygen and water; accelerated by heat, light, and contamination

- **Physical State**: Clear, colorless liquid; may appear slightly turbid

Task Requirements (Time-limited)

Task 1: Identify Oxidizer Hazards and Incompatibilities (Time limit: 30 seconds)
Instructions: "Using the provided safety information sheet, identify the primary hazards and incompatible materials for this oxidizing substance."

Correct Response Elements (must identify at least 4 of 6):

1. Strong oxidizer - can intensify fire

2. May cause combustion of combustible materials on contact

3. Incompatible with organic materials, reducing agents, and combustible materials

4. Incompatible with metals, acids, and bases

5. Decomposition releases oxygen, increasing fire risk

6. Contact with combustible materials may cause ignition

Task 2: Identify Immediate Response Actions (Time limit: 35 seconds)
Instructions: "Identify the immediate response actions required for this oxidizer leak."

Correct Response Elements (must identify at least 4 of 6):

1. Remove or isolate combustible materials from contact with oxidizer

2. Ensure adequate ventilation (oxygen release hazard)

3. Wear appropriate PPE (chemical-resistant gloves, goggles, face shield)

4. Absorb spill with inert, non-combustible absorbent material

5. Do NOT use organic absorbents (paper towels, sawdust, etc.)

6. Place contaminated materials in non-combustible waste container

Task 3: Determine Storage and Handling Corrections (Time limit: 25 seconds)
Instructions: "Identify the correct storage requirements to prevent future incidents."

Correct Response Elements (must identify at least 3 of 5):

1. Store in tightly closed container in cool, well-ventilated area

2. Protect from light and heat

3. Store away from combustible materials and reducing agents

4. Use vented caps or periodically release pressure buildup

5. Inspect containers regularly for signs of decomposition or pressure buildup

Scoring Rubric
|------------------|-------|----------|

Information Search Time Measurement
- **Start point**: Moment scenario description is presented

- **End point**: Moment participant correctly identifies oxidizing hazard classification

- **Recording method**: Digital timer with millisecond precision

- **Acceptable range**: 10-45 seconds (based on pilot testing)

General Evaluation Procedures

Evaluator Training
All evaluators underwent standardized training including:

1. Review of all scenario materials and scoring rubrics

2. Practice scoring with 10 pilot test recordings

3. Inter-rater reliability assessment (target ICC ≥ 0.85)

4. Calibration session to ensure consistent interpretation of scoring criteria

Participant Instructions
Before each scenario, participants received standardized instructions:

1. "You will be presented with a chemical incident scenario"

2. "Read the scenario carefully and use the provided safety information sheet (ESS or SDS) to answer the questions"

3. "Answer as quickly and accurately as possible"

4. "Your response time and accuracy will be recorded"

5. "There is no penalty for asking clarification questions before starting"

Data Recording
For each participant and scenario, the following data were recorded:

1. **Information search time**: Time from scenario presentation to first correct action identification (milliseconds)

2. **Task accuracy**: Number of correct response elements identified / total required elements (percentage)

3. **Overall scenario score**: Composite score based on rubric (0-100 scale)

4. **Evaluator notes**: Qualitative observations of participant approach and decision-making process

Quality Control
To ensure data quality and reliability:

1. All sessions were video recorded (with participant consent)

2. 20% of sessions were independently scored by a second evaluator

3. Discrepancies >10 points triggered review and consensus scoring

4. Inter-rater reliability was monitored throughout data collection (ICC maintained ≥0.91)

Statistical Analysis of Task-based Data

Primary Outcomes

1. **Emergency Response Accuracy**: Composite score across all four scenarios (0-100 scale)

2. **Information Search Time**: Mean time to identify first correct action across all scenarios (seconds)

3. **Escalation Accuracy**: Correct identification of reporting and communication protocols (percentage)

Analysis Methods

- Independent t-tests for continuous outcomes (search time)

- Chi-square tests for categorical outcomes (accuracy thresholds)

- Risk ratios and risk differences for binary outcomes

- Cohen's d for effect size estimation

- Bonferroni correction for multiple comparisons ($\alpha = 0.05/3 = 0.017$)

Sample Size Justification

Based on pilot data (n=10), we estimated:

- Mean search time: ESS 15±3 seconds, SDS 28±5 seconds

- Expected effect size: Cohen's d ≈ 3.0

- Required sample size: 40 per group (80% power, α=0.017 after Bonferroni correction)

Supplementary References

[4] Hazard Communication Standard: Safety Data Sheets; OSHA Brief DSG BR-3514; Occupational Safety and Health Administration (OSHA): Washington, DC, USA, 2012.

[2] World Health Organization (WHO). Laboratory Biosafety Manual, 3rd ed.; WHO/CDS/CSR/LYO/2004.11; World Health Or-ganization: Geneva, Switzerland, 2011.

[47] National Institute for Occupational Safety and Health (NIOSH). NIOSH list of antineoplastic and other hazardous drugs in healthcare settings, 2014. Available online: https://www.cdc.gov/niosh/docs/2014-138/pdfs/2014-138_v3.pdf (accessed on 5 November 2025).

[48] Intrusion warning and assessment method for site safety enhancement. Available online:https://www.sciencedirect.com/science/article/pii/S0925753515003331?via%3Dihub (accessed on 5 November 2025).
